# Deep Eutectic Solvent Formulations and Alginate-Based Hydrogels as a New Partnership for the Transdermal Administration of Anti-Inflammatory Drugs

**DOI:** 10.3390/pharmaceutics14040827

**Published:** 2022-04-10

**Authors:** Sónia N. Pedro, Maria S. M. Mendes, Bruno M. Neves, Isabel Filipa Almeida, Paulo Costa, Inês Correia-Sá, Carla Vilela, Mara G. Freire, Armando J. D. Silvestre, Carmen S. R. Freire

**Affiliations:** 1 Department of Chemistry and CICECO-Aveiro Institute of Materials, University of Aveiro, 3810-193 Aveiro, Portugal; soniapedro@ua.pt (S.N.P.); msilvinamm@ua.pt (M.S.M.M.); cvilela@ua.pt (C.V.); armsil@ua.pt (A.J.D.S.); 2 Department of Medical Sciences and Institute of Biomedicine-iBiMED, University of Aveiro, 3810-193 Aveiro, Portugal; bruno.neves@ua.pt; 3 Associate Laboratory i4HB, Institute for Health and Bioeconomy, Faculty of Pharmacy, University of Porto, 4050-313 Porto, Portugal; ifalmeida@ff.up.pt (I.F.A.); pccosta@ff.up.pt (P.C.); 4 UCIBIO, Applied Molecular Biosciences Unit, MedTech-Laboratory of Pharmaceutical Technology, Faculty of Pharmacy, University of Porto, 4050-313 Porto, Portugal; 5 Department of Plastic, Aesthetic, Reconstructive and Aesthetic Surgery, Centro Hospitalar de S. João, 4200-319 Porto, Portugal; inescsa@gmail.com

**Keywords:** alginate, biopolymers, deep eutectic solvents, drug delivery systems, nonsteroidal anti-inflammatory drugs, solubility, skin permeation

## Abstract

The transdermal administration of nonsteroidal anti-inflammatory drugs (NSAIDs) is a valuable and safer alternative to their oral intake. However, most of these drugs display low water solubility, which makes their incorporation into hydrophilic biopolymeric drug-delivery systems difficult. To overcome this drawback, aqueous solutions of bio-based deep eutectic solvents (DES) were investigated to enhance the solubility of ibuprofen, a widely used NSAID, leading to an increase in its solubility of up to 7917-fold when compared to its water solubility. These DES solutions were shown to be non-toxic to macrophages with cell viabilities of 97.4% (at ibuprofen concentrations of 0.25 mM), while preserving the anti-inflammatory action of the drug. Their incorporation into alginate-based hydrogels resulted in materials with a regular structure and higher flexibility. These hydrogels present a sustained release of the drug, which is able, when containing the DES aqueous solution comprising ibuprofen, to deliver 93.5% of the drug after 8 h in PBS. Furthermore, these hydrogels were able to improve the drug permeation across human skin by 8.5-fold in comparison with the hydrogel counterpart containing only ibuprofen. This work highlights the possibility to remarkably improve the transdermal administration of NSAIDs by combining new drug formulations based on DES and biopolymeric drug delivery systems.

## 1. Introduction

Nonsteroidal anti-inflammatory drugs (NSAIDs) represent one of the most commonly prescribed medications for pain and inflammation treatment [1]. The key effect of NSAIDs is primarily linked to their ability to block specific prostaglandins synthesis by the inhibition of cyclooxygenase enzymes (COX-1 and COX-2) [1]. This inhibition also plays a major role in the side-effects associated with their oral intake [2]. The inhibition of COX-2 plays a central role anti-inflammatory and analgesic effects of these drugs; however, its inhibition also impacts cardiovascular health with chronic drug usage. On the other hand, the inhibition of COX-1 is responsible for severe gastrointestinal ulceration and renal toxicity. Although the gastrointestinal safety of these drugs can be improved by association with phospholipids or the concomitant administration of gastroprotective pharmaceuticals, such as proton pump inhibitors, the cardio-nephrotoxic side-effects are still substantial [2]. Considering the chronic use of NSAIDs, the transdermal administration of these drugs can be an advantageous alternative, with considerable efficacy and safety [3]. Studies have shown that NSAIDs administered by this vein can permeate across skin [4,5]. Following this, numerous formulations of NSAIDs have become available, including creams, gels, patches and solutions (lotions), which have mainly been used for musculoskeletal pain conditions [6]. However, the formulation of NSAIDs in these systems is hindered by their low-water solubility, requiring the use of high contents of organic solvents, such as ethanol, to solubilize them and improve their permeation [7].

In an attempt to avoid the use of organic solvents and to improve the delivery of low-water-soluble molecules, eutectic mixtures have been explored as alternative pharmaceutical solvents and permeation enhancers [8,9,10]. Among these eutectic mixtures, deep eutectic solvents (DES) can be highlighted, due to their strong deviations from the ideal solid–liquid phase behavior that can result in liquids below room temperature [11,12]. These mixtures have shown to be competitive solvents and permeators compared to common organic solvents and co-solvents. By using DES, it is possible to improve the solubility of anti-inflammatory drugs (such as ibuprofen, naproxen, ketoprofen) [13] and paracetamol [14], but also to enhance the permeation of these drugs across skin. Nevertheless, the use of hydrophobic DES to this purpose poses the same issues as those associated with the use of organic compounds concerning the development of biopolymer-based drug delivery systems, such as hydrogels, which are typically based on hydrophilic biopolymers, such as polysaccharides [15]. Therefore, the study and application of water-soluble DES needs to be further explored as an approach to the simultaneous solubilization of NSAIDs and their incorporation in hydrogel-based drug-delivery systems.

Hydrogels possess a high water content and a 3D microstructure that can make them valuable as delivery systems for transdermal drug administration, ensuring simplicity of application, with a significant minimization of side effects [15]. One example of a biopolymer that is commonly used in these systems is alginate [16]. Alginate is a readily available polysaccharide, mainly extracted from brown seaweed. Since alginate is non-immunogenic, non-toxic and presents good biocompatibility, it has been extensively explored in the biomedical field for wound healing [17,18], tissue engineering [19] and drug-delivery purposes [20,21]. Alginate-based hydrogels are commonly formed by physical or chemical cross-linking and have been explored for the continuous and local administration of small pharmaceutical ingredients, resulting in the sustained and controlled release of these drugs from the hydrogel network [17,18]. Such hydrogels have proved to be effective and safe for skin applications, with low levels of associated skin irritancy, as demonstrated for the transdermal administration of the antihypertensive drug prazosin hydrochloride [22]. Furthermore, alginate-based hydrogels have been also studied for the incorporation of NSAIDs, such as ibuprofen; however, these present a low loading capacity and homogeneity [23]. This limitation has increased the search for strategies to improve the compatibility of hydrogels and hydrophobic drugs. Even though the combination of alginate and DES is promising, it has scarcely been explored, with only one study focused on the use of DES to favor the encapsulation of curcumin into alginate/chitosan beads for oral administration [24]. To the best of our knowledge, there are no previous reports on the combination of alginate hydrogels and DES for the improvement of the transdermal delivery of pharmaceutical ingredients.

Taking advantage of the properties of both DES and alginate, in this study, their partnership for the transdermal administration of hydrophobic molecules, such as NSAIDs, using ibuprofen as a model drug, was explored. We demonstrated the possibility to enhance the solubility and stability of ibuprofen by using aqueous solutions of DES, and the improvement in its skin permeation when incorporated into an alginate solid hydrogel system.

## 2. Materials and Methods

### 2.1. DES Preparation and Characterization

In this work, we studied the DES arginine:glycerol (Arg:Gly) to solubilize ibuprofen (Alfa Aesar, Haverhill, MA, USA, 99%). This DES was prepared by mixing arginine (Panreac, Barcelona, Spain, 99%) with glycerol (Sigma-Aldrich, St. Louis, MO, USA, ≥99.5%) in a 1:4 molar ratio. This mixture was placed in sealed glass vials with constant stirring while heated (maximum temperature of 85 °C), until a homogeneous transparent liquid was obtained. After being kept at the maximum temperature for 1 h, the mixture was allowed to return to room temperature. Aqueous solutions (0–60% (*w*/*w*)) of Arg:Gly were prepared from this neat DES by addition of the proper water amount.

To infer about the integrity of the Arg:Gly components in the DES, ^1^H and ^13^C nuclear magnetic resonance (NMR) spectroscopy was carried out. The spectra were recorded using a Bruker Avance 300 spectrometer (Bruker Corporation, Billerica, MA, USA) operating at 300.13 MHz for ^1^H NMR, and at 75.47 MHz for ^13^C NMR. The mixtures were dissolved in deuterated water using trimethylsilyl propanoic acid (TMSP) as an internal reference. The interactions between the components of the DES were analyzed by Fourier Transform Infrared-Attenuated Total Reflectance (FTIR-ATR) spectroscopy. The spectra of arginine, glycerol and the Arg:Gly (1:4) were obtained on a Perkin Elmer spectrometer (Perkin-Elmer Inc., Waltham, MA, USA) equipped with a single horizontal Golden Gate ATR cell and a diamond crystal. Data were recorded by the accumulation of 32 scans performed at room temperature in the range of 4000–400 cm^−1^, with a resolution of 4 cm^−1^ and an interval of 1 cm^−1^. All spectra were subtracted against background air spectrum and recorded in transmittance mode.

### 2.2. Solubililty Measurements

To determine the drug solubility, ibuprofen was added in excess to 2.0 g of arginine:glycerol (1:4) aqueous solutions (0–60% (*w*/*w*) of DES) and pure water. These solutions were placed in sealed glass vials and allowed to equilibrate in a specific aluminium disk at constant temperatures (room (25 °C) and body (37 °C) temperatures) and under stirring (900 rpm) over 72 h. The solubility of ibuprofen was studied in the range of 0 to 60% (*w*/*w*) of Arg:Gly in water, given the high viscosity of the mixtures above this concentration. After saturation of each DES aqueous solution (0–60% *w*/*w*), the samples were centrifuged, and an aliquot of the supernatant was collected and diluted in distilled water. The sample was then carefully filtered with a 0.45 μm syringe filter to remove any solid from the liquid phase and subsequently analysed by high-performance liquid chromatography with diode-array detection (HPLC-DAD) (Shimadzu, model PROMINENCE, Kyoto, Japan). The HPLC quantifications were performed in isocratic mode with an analytical C18 reversed-phase column (250 × 4.60 mm), Kinetex 5 μm C18 100 Å, from Phenomenex. The mobile phase consisted of 45% (*v*/*v*) of acetonitrile and 55% (*v*/*v*) of ultra-pure water with 0.3% (*v*/*v*) of ortho-phosporic acid. The separation was conducted using an injection volume of 10 μL at a flow rate of 0.8 mL·min^−1^ and operated at 25 °C. The wavelength was set at 264 nm and each sample was analysed at least in triplicate. Calibration curves were obtained using ibuprofen dissolved in the mobile phase. Under the referred conditions, ibuprofen displays a retention time of 15.2 min.

### 2.3. Drug Stability Storage

To evaluate the stability of ibuprofen in the novel formulations, the drug was dissolved in water and in the Arg:Gly aqueous solution (60% (*w*/*w*) of DES) below the solubility limit. An initial aliquot of each solution was then analyzed by HPLC-DAD to determine the drug content in each formulation (T_0_). The solutions were then kept at 25 and 37 °C and at 75–80% relative humidity for 30 days and protected from light. After this period, new aliquots were collected and quantified by HPLC-DAD using the previously described method. Two independent studies were conducted, and each sample was analysed at least in triplicate.

### 2.4. Biological Activity

#### 2.4.1. Cell Culture

Murine Raw 264.7 macrophages (ATCC number: TIB-71) were cultured in Dulbecco’s Eagle Medium (DMEM) containing 4.5 mg·mL^−1^ glucose, 4 mM *L*-glutamine, 1.5 mg·mL^–1^ sodium bicarbonate and supplemented with 10% non-inactivated fetal bovine serum (FBS), 100 IU·mL^−1^ penicillin, and 100 μg·mL^−1^ streptomycin. Cells were incubated in a humidified atmosphere of 95% of air and 5% of CO_2_ at 37 °C and were used after reaching up to 80% confluence.

#### 2.4.2. Cell Viability Assays

The impact of ibuprofen solubilized in Arg:Gly aqueous solutions on macrophage cells viability was evaluated by the resazurin assay. Firstly, 4 × 10^4^ of Raw 264.7 cells/well were plated on a 96 well plate and let to stabilize overnight. Then, pure ibuprofen, the Arg:Gly aqueous solution (60% (*w*/*w*) of DES in water and ibuprofen solubilized in the Arg:Gly aqueous solution, in the concentrations range of 0.01–4 mM, were added to the cell cultures for 24 h. After this, resazurin (Sigma-Aldrich, cell culture grade) was added to the cells during the last hour of incubation, achieving a final concentration of 50 μM. Lastly, the absorbance was measured at 570 and 600 nm in a BioTek Synergy HT spectrophotometer (Biotek Instruments, Winooski, VT, USA). The reported data are from three biological independent experiments conducted in duplicate for each condition and the results were expressed as the average cell viability ± standard deviation (SD). The aqueous solutions used in the assay were previously sterilized by filtration.

#### 2.4.3. Anti-Inflammatory Assays

The anti-inflammatory action of ibuprofen solubilized in water and in the Arg:Gly aqueous solution was evaluated by the ability to inhibit the lipopolysaccharide (LPS)-induced nitric oxide (NO) production in macrophages. The NO production was measured by a colorimetric assay with the Griess reagent (0.1% (*w*/*v*) *N*-(1-naphthyl)ethylenediamine dihydrochloride (Sigma-Aldrich, ≥98) and 1% (*w*/*v*) sulfanilamide (Sigma-Aldrich, ≥98) containing 5% (*w*/*v*) H_3_PO_4_) (Sigma-Aldrich, ≥85 wt.% in H_2_O), that aimed to detect the accumulation of nitrite in the culture supernatants. To this purpose, the cells were plated at 3 × 10^5^ cells/well in 48-well culture plates, allowed to stabilize for 12 h, and then incubated as previously described with non-cytotoxic concentrations of formulations. Incubations with the culture medium (control), ibuprofen (250 μM), ibuprofen (250 μM) in the DES aqueous solution (60% (*w*/*w*) of DES in water) were performed over 24 h. After incubation, 100 μL of culture supernatants were collected and mixed with an equal volume of the Griess reagent and kept during 15 min in the dark. The absorbance of these samples was measured at 550 nm, using a standard spectrophotometer BioTek Synergy HT (Biotek Instruments, Winooski, VT, EUA). Multiple group comparisons were executed by One-Way ANOVA analysis using GraphPad Prism, version 6.01 (GraphPad Software, San Diego, CA, USA).

### 2.5. Incorporation of Aqueous Solutions of DES in the Alginate Hydrogel

#### 2.5.1. Preparation of the Alginate-Based Hydrogels

Four different solid hydrogels were prepared using an alginate aqueous solution with a concentration of 4% (*w*/*v*), namely, alginate hydrogels without the drug (Alg), with ibuprofen (Alg-Ibu), with the Arg:Gly aqueous solution (Alg-DES) (60% (*w*/*w*) of DES) and with ibuprofen solubilized in the Arg:Gly aqueous solution (Alg-(DES + Ibu)). Ibuprofen was previously solubilized in the Arg:Gly aqueous solution to obtain hydrogels loaded with 20 mg of ibuprofen. For the hydrogels containing ibuprofen solubilized in the Arg:Gly aqueous solution and the ones only with the Arg:Gly aqueous solution, the solvent represented 3% (*w*/*v*) of the totality of the hydrogel. In the Alg-Ibu hydrogels, due to the drug’s low-water solubility, the DES amount was replaced by a 0.1 M aqueous NaOH solution [25]. Firstly, alginate was dissolved in water under continuous stirring (500 rpm) until a homogeneous solution was obtained. The Arg:Gly aqueous solution, ibuprofen, and ibuprofen solubilized in the Arg:Gly aqueous solution were added to the corresponding alginate solutions during the biopolymer solubilization process. These solutions were then placed in a sonication bath (ElmaSonic S 300, Singen, Germany) for removal of the entrapped air bubbles. Finally, samples were poured into circular molds and crosslinked for 1 h at room temperature, by osmosis, using a 10% (*w*/*w*) calcium chloride (CaCl_2_) solution.

For the hydrogels containing ibuprofen, the drug incorporation efficiency was calculated based on the total amount of ibuprofen added to the formulations and the amount retained in the hydrogels after their preparation, according to the following equation:

$$\mathrm{Incorporation}\;\mathrm{efficiency}\%=\frac{Mass\;of\;ibuprofen\;in\;the\;hydrogel}{Total\;mass\;of\;ibuprofen}\times 100\%$$

The mass of ibuprofen retained in the hydrogel was calculated by subtracting the amount of ibuprofen dissolved in the CaCl_2_ solution during cross-linking of the hydrogel to the total amount initially added to the formulations.

#### 2.5.2. Evaluation of the Morphology and Mechanical Properties of the Hydrogels

The morphological analysis of the hydrogels loaded with ibuprofen and with the DES aqueous solution comprising ibuprofen was performed by scanning electron microscopy (SEM). For this analysis, the hydrogel samples were first frozen in a refrigerator at −80 °C for 24 h and then freeze-dried for 72 h at −85 °C and 0.01 mbar using a freeze dryer (Telstar, LyoQuest, Tokyo, Japan). Freeze-dried samples were coated with carbon using an EMITECH K950 coating system before the analysis. The micrographs of the cross-sections of the samples (obtained by cutting with a sharp razorblade) were acquired using a high-voltage microscope (HITACHI SU 70, Tokyo, Japan) operated at 4.0 kV. For determination of the porous sizes of the hydrogels, SEM micrographs were processed with ImageJ software (version 1.53 for Windows, 64-bit, free software, National Institutes of Health, Bethesda, MD, USA) and values are expressed as mean of 50 different measurements.

Mechanical properties of the hydrogels loaded with ibuprofen, Alg-Ibu and Alg-(DES + Ibu), and of Alg and Alg-DES (for comparison purposes), were evaluated through compressive assays performed using an Instron 5564 (Instron Corporation, Norwood, MA, USA) testing machine with Bluehill 3 software in compressive mode with a 50 N load cell. Circular samples with a 2-cm diameter and 4-mm gauge length were used. At least 5 replicates were tested for each sample. The corresponding compressive Young’s modulus (MPa) and the compressive stress at 30% (MPa) values were determined and expressed as the average ± SD. Multiple group comparisons were executed by One-Way ANOVA analysis using GraphPad Prism, version 6.01 (GraphPad Software, San Diego, CA, USA).

#### 2.5.3. Ibuprofen Dissolution Test

The Alg-Ibu and Alg-(DES + Ibu) solid hydrogels were immersed in 200 mL of a 0.01-M phosphate buffer (pH 7.4) solution. The dissolution was then carried out at 32.0 ± 1 °C and constant stirring (100 rpm). Aliquots of 1 mL were collected at specific timepoints (0, 5, 10, 30, 60, 90, 120, 180, 240, 300, 360, and 420 min) for a total of 8 h. The same volume of fresh buffer solution was added to maintain a constant volume. The dissolved ibuprofen in each aliquot was quantified by HPLC-DAD, at 264 nm, using the previously described method. The percentage of ibuprofen release at each time was determined based on the ratio of the amount of drug released and the total drug content in the hydrogels. Three replicates were performed for each sample.

#### 2.5.4. Skin Permeation Assays

Human abdominal skin samples were used for the permeation assays. These samples were obtained from women under the ages of 25–35 who were submitted to an abdominoplasty in Centro Hospitalar São João, Portugal. All patients signed the respective informed consent. The approval of the Ethics Committee of Hospital São João was also obtained for this procedure. After collection, the skin samples were transported under refrigerated conditions. Hypodermis was removed using a scalpel, and then the skin surface was washed, dried and frozen at −20 °C. Skin biopsies were obtained using a biopsy punch (30 mm diameter) to fit the Franz diffusion cell apparatus. The skin was placed in water at 65 °C for 80 s to separate the epidermis, as previously reported in the literature [26]. The experiments with epidermal membranes were conducted on glass Franz-type diffusion cells with a receptor volume of ca. 7 mL and a diffusional area of 1.77 cm^2^. An hydroalcoholic solution of phosphate buffered saline solution (PBS, pH 7.4) and ethanol (1:1) was used as the receptor media. The receptor compartment was maintained at 37 °C and under constant stirring. Alginate hydrogels with a load of 20 mg of ibuprofen were cut to fit the surface area (1.77 cm^2^) of the donor compartment and cover the entire epidermal surface. After 6 h, the receptor solution was withdrawn from the receptor compartment and the amount of permeated ibuprofen was quantified. The drug content was analyzed by HPLC-DAD, at 264 nm, using the method previously described for ibuprofen. The permeation assays were carried out in triplicate, and each aliquot was measured twice. Results were expressed as the average of permeated drug ± SD.

## 3. Results and Discussion

**Figure 1:**
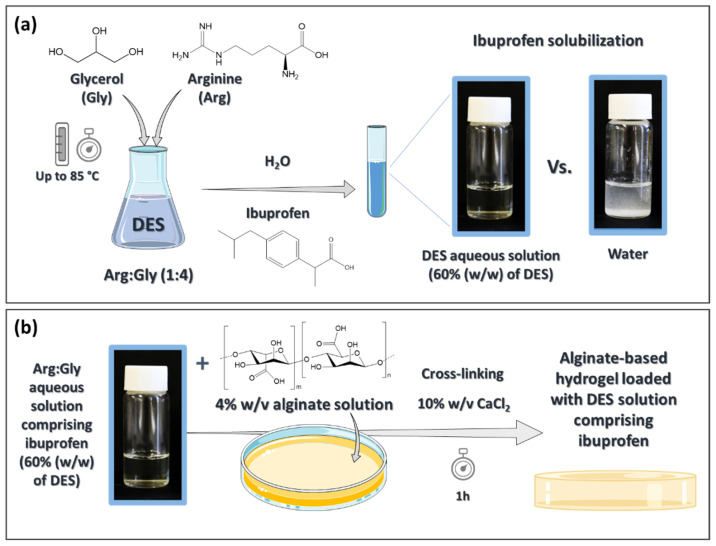
Schematic illustration of the preparation of a DES aqueous solution with higher solubilization ability for ibuprofen (**a**) and its incorporation in an alginate-based hydrogel (**b**) developed for the transdermal delivery of ibuprofen. Image made with Servier Medical Art and adapted by the authors according with Servier under the CC-BY 3.0 License (at https://smart.servier.com/, accessed on 23 January 2020).

NSAIDs, such as ibuprofen, present very low water solubility at room temperature (4.3 × 10^−5^ mol L^−1^ for ibuprofen) [27]. However, this solubility not only increases with temperature, it can also be increased by adding organic co-solvents to the aqueous solutions, such as ethanol, which are commonly used in commercial topical formulations [28,29]. Due to the negative effect of ethanol on skin, such as dryness, and its association with the development of several skin disorders (e.g., eczema, psoriasis) [30], the solubilization of ibuprofen and its stability in DES aqueous solutions was studied in this work. The components of the DES used herein, viz. glycerol and arginine, were selected due to their administration route and the intended efficacy improvement. Glycerol is widely used as a humectant in skin formulations [31], and arginine has been reported to down-regulate cytokine secretion and decrease the activity of metalloproteinases, helping to decrease inflammation [32]. This DES, Arg:Gly (1:4 molar ratio), was prepared by the heat-method and ibuprofen was added to the respective aqueous solution (Figure 1a); then, the drug solubility, stability, cytotoxicity and anti-inflammatory action were appraised. The DES formulation comprising ibuprofen (with 60% (*w*/*w*) of DES in water) was then incorporated in an alginate-based hydrogel (Figure 1b), which was characterized in terms of its morphology, mechanical properties, dissolution and permeation profiles through human skin. A hydrogel containing ibuprofen solubilized in a slightly alkaline aqueous solution was prepared and characterized for comparison. These characterizations were performed to evaluate the potentialities of the partnership between DES solutions and alginate hydrogels to improve the transdermal delivery of ibuprofen.

### 3.1. DES Characterization

Firstly, the structure of the hydrogen-bond donor/acceptors was assessed after the preparation of the DES containing Arg:Gly. This was evaluated by ^1^H and ^13^C NMR spectroscopy, as depicted in the Supplementary Materials (Figures S1a and S2b, respectively). The recorded spectra show that both components maintain their structure after the DES preparation. Such conclusions can be drawn due to the similarity of the resonances observed for Arg:Gly to those predicted for the single components’ spectra. Afterwards, the DES formation and the interaction between the components was confirmed by FTIR-ATR spectroscopy (Figure S2). By comparing the FTIR-ATR spectra of the pure compounds with that of the DES, it is possible to observe that the DES spectrum is more similar to that of glycerol. This can be expected since Arg:Gly was prepared in a 1:4 molar ratio. Specifically, it is possible to verify that, in the spectrum of glycerol, there is an absorption band at 3279 cm^−1^, which corresponds to the hydroxyl groups’ (O–H) stretching vibration and the peaks corresponding to the C–H stretching in the region of 2812–2980 cm^−1^ [33]. For arginine, O–H and N–H stretching vibrations appear at the region of 2940–3324 cm^−1^, while the absorption bands observed at 1550 cm^−1^ and 1560 cm^−1^ correspond with the C–O vibration and the C=O stretching of the carbonyl group, respectively [34]. In the DES spectrum, the establishment of hydrogen–bond interactions, among others, between both components leads to a slight deviation in the vibration of the N–H and O–H groups to 3255 cm^−1^, and of the peaks’ corresponding to the C–H stretching to values in the region of 2838–2970 cm^−1^. This is also verified for the C–O vibration and the C=O stretch of the carboxyl group of arginine, which are found at 1565 cm^−1^ and 1634 cm^−1^, respectively, in the DES spectrum.

### 3.2. Ibuprofen Solubility in DES Aqueous Solutions

**Figure 2:**
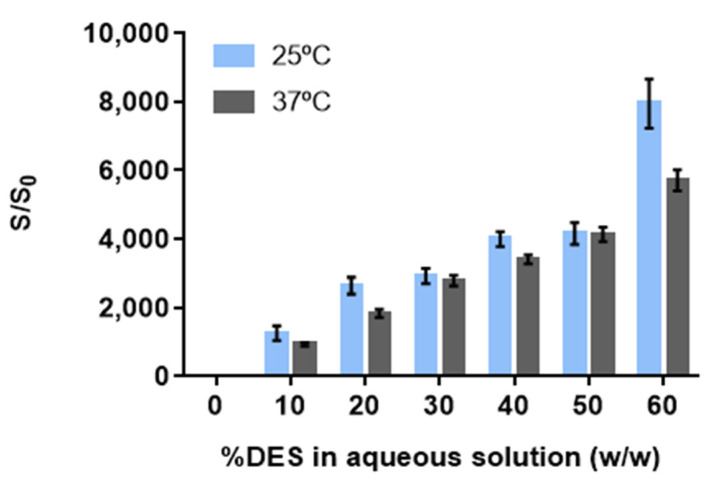
Solubility enhancements for ibuprofen in arginine:glycerol aqueous solutions achieved at both room and body temperatures. The results are expressed as the mean ± SD of three independent experiments.

Aqueous solutions of Arg:Gly (1:4) were tested up to 60% (*w*/*w*) of DES given the high viscosity of solutions above this concentration. To understand the solubilization mechanism of ibuprofen, and which Arg:Gly concentration is the most effective to the intended purpose, the solubility of the drug at both room (25 °C) and human body (37 °C) temperatures (values provided in Supplementary Information, Table S1) was investigated in aqueous solutions with different DES percentages. The solubility enhancements presented in Figure 2 are calculated from the ratio (S/S_0_) between the solubility of ibuprofen in each Arg:Gly aqueous solution (S) and its solubility in water (S_0_) at the same temperature.

The solubility of ibuprofen in water increases from (2.163 ± 0.158) × 10^−5^ mol∙dm^−3^ up to (3.186 ± 0.177) × 10^−5^ mol∙dm^−3^ from room to body temperature (Table S1). However, these concentrations are still inadequate for therapeutic purposes. The dosage of ibuprofen in common commercial formulas is generally 200 mg, administered every 6 h [35]. However, the quantity required for therapeutic purposes in an adult is only approximately 20 mg. The usually commercialized forms possess more than 10-fold this amount, not only due to their first pass metabolism and poor absorption, but also due to the poor aqueous solubility of the drug. This limited solubility and the rate of dissolution from the currently available solid forms leads to poor bioavailability; therefore, higher doses need to be administered to reach the therapeutic dosage contributing to the increase in some of the unwanted adverse effects [2].

For the Arg:Gly aqueous solutions studied here, the solubility of the drug increases proportionally to the increment of the Arg:Gly percentage (*w*/*w*) in water. This monotonic behavior reveals that the Arg:Gly solubilization ability is in agreement with a co-solvency mechanism [36]. When using 60% (*w*/*w*) of Arg:Gly in the aqueous solution, (1.71 ± 0.02) × 10^−1^ mol∙dm^−3^ of ibuprofen are solubilized; this amount is equivalent to 46.92 mg, which is 2-fold the therapeutic dosage needed [35]. This represents a notable 7917-fold increase in the drug solubility when compared to its solubility in water at the same temperature (Figure 2). At body temperature, since the drug solubility is higher, this value was increased up to (1.82 ± 0.03) × 10^−1^ mol∙dm^−3^ (Table S1), meaning a 5705-fold increase in the solubility of ibuprofen when compared to its water solubility at 37 °C (Figure 2). Since the amount of ibuprofen that can be solubilized in the Arg:Gly aqueous solutions shows a monotonic increase, maximum solubility was not fully achieved. Higher drug amounts are expected to be solubilized above 60% (*w*/*w*) of DES in water; however, these values were not determined due to the viscosity of the mixtures above these concentrations, as previously stated. Nevertheless, the solubility enhancements obtained using this DES solution surpass the increases in solubility achieved by the use of aqueous solutions of co-solvents, such as propylene glycol and poly(ethylene glycol) (PEG 300), that allow for a 400-fold and 1500-fold increase in the water solubility of ibuprofen at room temperature, when using 80% (*w*/*w*) of each co-solvent [37]. Eutectic mixtures, and particularly DES, have also been studied to improve the solubility of ibuprofen in water and in neat DES, for transdermal applications [14,38]. For example, a 4-fold increase in water solubility was achieved with the mixture menthol:camphor (1:1) [38]. However, the DES menthol:camphor presents itself as having low water solubility, representing a problem for its application in water-rich matrices. Solubility enhancements of more than 3810-fold have also been verified for neat DES, such as cholinium chloride:propanediol (1:5); however, this enhancement is highly reduced when less than 75% (*w*/*w*) of DES is used in an aqueous solution [14].

In sum, by applying Arg:Gly aqueous solutions, it is not only possible to obtain higher solubility enhancements than the DES previously reported in the literature [38] and the previously mentioned co-solvents [37], but also, to use less co-solvent (in this case, DES) in the final formulation. Therefore, the aqueous solution with 60% of Arg:Gly (*w*/*w*) was selected for the further study. 

### 3.3. Ibuprofen Stability in the DES Aqueous Solutions

**Figure 3:**
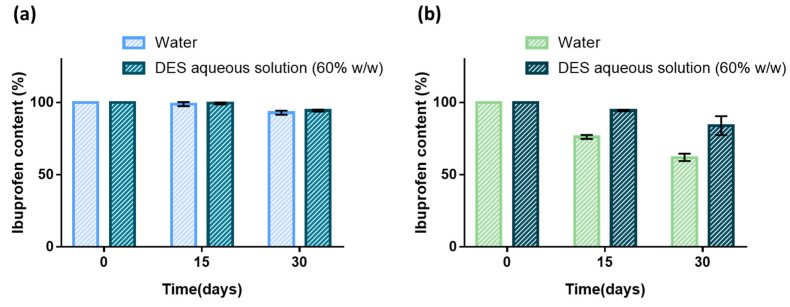
Effect of the solvent in the stability of ibuprofen at 25 °C (**a**) and 37 °C (**b**) over a period of 30 days. The results are expressed as the mean ± SD of three independent experiments.

The stability of ibuprofen in aqueous solution containing 60% (*w*/*w*) of DES was investigated at 25 and 37 °C. The content of ibuprofen solubilized in the Arg:Gly aqueous solution, and in water for comparison, was analyzed each 15 days for both temperatures, as shown in Figure 3.

When stored at 25 °C, ibuprofen presents high stability in both water and the DES aqueous solution, making it possible to quantify 93–95% of ibuprofen after 30 days of storage (Figure 3a). For higher temperatures, namely, 37 °C, the stability of ibuprofen in water decreases, with 76.2 ± 1.0% of ibuprofen being quantified in solution after 15 days and only 62.1 ± 1.8% after 1 month. The use of DES aqueous solutions clearly improved the stability of ibuprofen when the drug was submitted to higher temperatures, as depicted in Figure 3. After 15 days at 37 °C, the drug content was 94.5 ± 0.4%, and there was still 84.1 ± 4.5% of ibuprofen in the formulation after 1 month.

It has been reported [39] that when ibuprofen is solubilized in 0.9% sodium chloride or 5% dextrose aqueous solutions, more than 92% of its initial concentration is retained when stored for 14 days at 4 °C. Liposomal formulations of ibuprofen were also reported to be stable under similar conditions during the same period [40]. Thus, it was possible not only to improve the ibuprofen solubility by using the aqueous DES as a pharmaceutical co-solvent, but also to improve the stability of the drug without requiring the use of additional excipients or storage under refrigerated conditions.

### 3.4. Cytotoxicity and Anti-Inflammatory Activity of DES-Based Formulations Containing Ibuprofen

**Figure 4:**
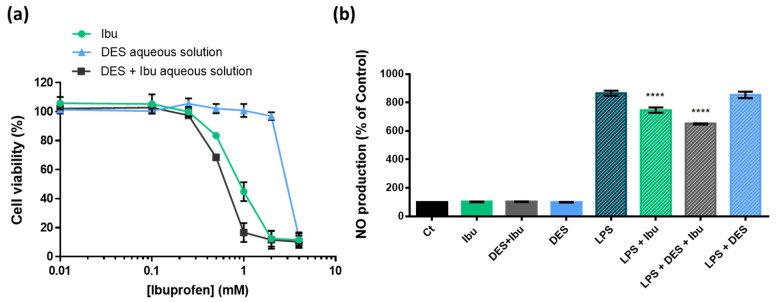
Effect of ibuprofen, Arg:Gly aqueous solution (60% *w*/*w*) and ibuprofen solubilized in Arg:Gly aqueous solution on the (**a**) cell viability profile of Raw 264.7 macrophages, measured by the metabolic conversion of resazurin; and on the (**b**) anti-inflammatory action evaluated by the NO production of Raw 264.7 macrophages. Results were expressed relative to the control as mean ± SD of three independent biological experiments. Statistically significant differences were using one-way ANOVA (**** = *p* ≤ 0.0001).

The cytotoxicity of ibuprofen that is solubilized in the Arg:Gly aqueous solution (60% *w*/*w*), of ibuprofen and of the Arg:Gly aqueous solution for comparison was evaluated in cells that were relevant for the intended application, namely, macrophages. In Figure 4, the cytotoxicity results obtained for a concentration range of 0.01–4.00 mM of ibuprofen are presented. The Arg:Gly aqueous solution (60% *w*/*w*) was tested in the same concentrations as the one with the Arg:Gly, comprising ibuprofen for comparison purposes.

As depicted, the Arg:Gly aqueous solution is not cytotoxic towards macrophages at concentrations up to 2.0 mM. This cytotoxicity profile is expected, since the DES’ components and the initial DES concentration were carefully selected to be non-toxic, in accordance with previous findings in the literature [41,42]. Although there are no data in the literature about the cytotoxicity of the DES used in the present study, the cytotoxicity of other DES comprising several alcohols and cholinium chloride has been reported in the literature for human skin cells (immortalized human epidermal keratinocytes (HaCaT)) [43], and, in most cases, these were shown to be harmless even at concentrations up to 500 μg mL^−1^ (above the ones tested in this work). The cytotoxicity profile of ibuprofen solubilized in the Arg:Gly aqueous solution is generally similar to that of the aqueous solutions of ibuprofen, and non-toxic to macrophages in concentrations below 0.5 mM. This dose–response profile, obtained for ibuprofen solubilized in both water and in the Arg:Gly aqueous solution (60% *w*/*w*), is in accordance with previous results for ibuprofen solubilized in water towards the same cell line and range of concentrations [44]. Therefore, our results show that the Arg:Gly aqueous solution containing ibuprofen in concentrations up to 0.5 mM is safe to be applied in transdermal drug delivery administration.

**Figure 5:**
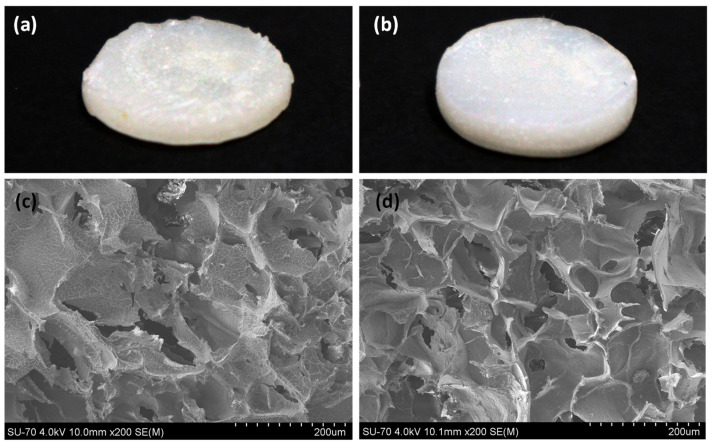
(**a**,**b**) Visual aspect of alginate-based hydrogels with (**a**) ibuprofen (Alg-Ibu) and (**b**) with DES aqueous solutions comprising ibuprofen (Alg-(DES + Ibu)) and (**c**,**d**) the corresponding cross-section SEM micrographs.

To find the influence of these formulations on the anti-inflammatory activity of ibuprofen, their effect on the NO and LPS-induced production in macrophages was analyzed (Figure 5). Macrophages can increase NO secretion after LPS stimulation in vitro, providing a good model to evaluate the anti-inflammatory potential of drug formulations [45]. If a given drug formulation presents anti-inflammatory action, the NO production after the LPS stimulation will decrease. Figure 5 shows that the Arg:Gly aqueous solution per se does not present the ability to inhibit NO production. Ibuprofen treatment decreased LPS-triggered NO production by macrophages. This may be attributed to its inhibition of NF-κB, a transcription factor essential for iNOS expression. However, this inhibition is relatively modest, and the anti-inflammatory activity of ibuprofen mostly relies on its inhibition of the COX-2 enzyme [2]. Surprisingly, when ibuprofen is solubilized in the Arg:Gly aqueous solution, a synergistic effect can be observed, and it is possible to obtain a statistically significant decrease in NO production when compared to both components separately. By solubilizing ibuprofen in the Arg:Gly in aqueous solution, it was verified that this formulation does not jeopardize the anti-inflammatory action of the drug and allows for a reduction in inflammation by providing a small increase in the therapeutic activity of the drug.

### 3.5. Incorporation of Aqueous Solutions of DES in an Alginate Hydrogel

The DES aqueous solution (60% (*w*/*w*) of DES in water) has shown promising ability to enhance the solubility and stability of ibuprofen, while preserving its anti-inflammatory action without cytotoxicity towards macrophages. To deliver this formulation with improved efficacy, the incorporation of this ibuprofen formulation into an alginate-based hydrogel was investigated. The effect of the use of Arg:Gly-based DES on the morphologic and mechanical properties of the biopolymer-based system, and on ibuprofen’s dissolution in a buffer solution and permeation across human skin, was evaluated.

The alginate hydrogels with ibuprofen solubilized in a slightly alkaline aqueous solution (Alg-Ibu) and ibuprofen in the Arg:Gly aqueous solution (Alg-(DES + Ibu) present distinct visual aspects and morphologies, as depicted in Figure 5.

While Alg-Ibu hydrogels exhibited a rougher and waver surface (Figure 5a), Alg-(DES + Ibu) ones showed a relatively smoother surface (Figure 5b). To corroborate these visual observations, the cross-sections of both freeze-dried hydrogels (Alg-Ibu and Alg-(DES + Ibu)) were also examined by SEM. The obtained micrographs show that both hydrogels have a porous 3D structure (Figure 5c,d). However, the pore shapes of the Alg-Ibu hydrogel are more irregular than those of Alg-(DES + Ibu), which present more clearly defined walls and a considerably higher homogeneity. A similar trend was reported for Basiak et al. [46], where the microstructure of wheat starch films also showed an increase in the homogeneity and a smoother surface when glycerol (a component of the DES used in the present study) was added to the biopolymer-based system. Moreover, Alg-(DES + Ibu) hydrogel presented slightly smaller pores (112.24 ± 8.51 μm) than the Alg-(Ibu) counterpart (138.77 ± 9.30 μm).Additionally, when considering the micrograph of the Alg-Ibu cross-section (Figure 5c), is possible to notice a mild drug precipitation, which is not observed for the Alg-(DES + Ibu) systems (Figure 5d) and which can possibly justify the mechanical properties further discussed in this section. This observation is certainly due to the higher solubility of ibuprofen in the DES aqueous solutions, as previously described.

**Figure 6:**
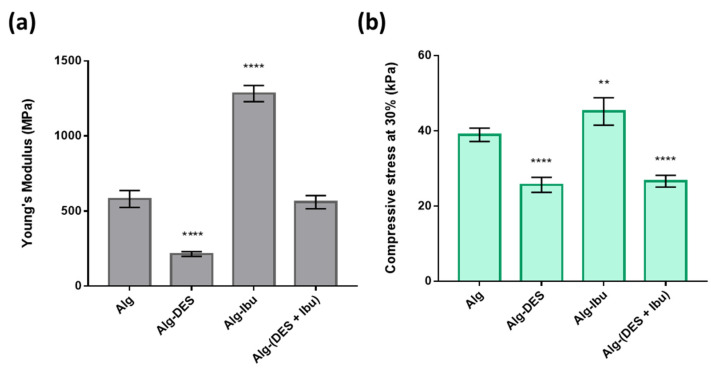
(**a**) Young’s modulus and (**b**) compressive stress at 30% of pure alginate (Alg), and alginate with DES solution (Alg-DES), ibuprofen (Alg-Ibu) and DES solutions containing ibuprofen (Alg-(DES + Ibu)) hydrogels obtained from the compressive tests. Values are presented as mean of five replicates and respective standard deviations. ** *p* < 0.0080, **** *p* < 0.0001 compared to the Alg-based hydrogel mechanical performance results.

The mechanical performance of the two solid hydrogels were studied by compressive tests. To better understand their performance, pure alginate hydrogels (Alg) and hydrogels with the DES solution (Alg-DES) were also tested for comparison purposes. The Young’s modulus (Figure 6a) and the compressive stress at 30% (Figure 6b) of the different samples were determined from the respective stress–strain curves.

The Alg hydrogel showed a Young’s modulus of 580 ± 50 kPa and a compressive stress at 30% of 39 ± 2 kPa. A lower Young’s modulus of 214 ± 14 kPa (Figure 5a) and a lower compressive stress at 30% of 26 ± 2 kPa (Figure 5b) was obtained for the Alg-DES hydrogel. These results reveal that the incorporation of the DES solution has a noticeable plasticizer effect, with a considerable impact on the mechanical properties of the Alg hydrogel network. This effect was previously observed for other biopolymers-based systems, for instance, for cellulose [47] and starch-based ones [48], comprising DES, such as cholinium chloride:glycerol and other cholinium and alcohol-based DES. In the present study, this can be obviously attributed to the effect of glycerol, which is known to present a plasticizer effect on biopolymers, and particularly on alginate hydrogels [49]. The use of glycerol-based DES not only promotes the formation of hydrogen-bonding between the biopolymer and the DES, but also decreases the biopolymer matrix’s strong intramolecular attraction, thereby increasing the interchain spacing, as see in previously reported alginate-based systems plasticized with glycerol [49,50]. This reduction in the interchain interactions of alginate, which can be attributed to the effect of glycerol from Arg:Gly, results in alginate hydrogels with lower rigidity.

However, the incorporation of ibuprofen solubilized in a slightly alkaline aqueous solution in the alginate hydrogel (Alg-Ibu) promoted an increase in the Young’s modulus and on the compressive stress at 30%, with values of 1283 ± 47 kPa and 45 ± 3 kPa, respectively (Figure 6a,b). Therefore, the incorporation of the drug in the hydrogel network turns it into a more rigid and brittle system. These findings have also been reported for the incorporation of ibuprofen into gellan gum-based hydrogels [51].

The Alg-(DES + Ibu) displayed a Young’s modulus of 559 ± 38 kPa and compressive stress values of 27 ± 1 kPa. Regarding the Young’s modulus, the obtained value is similar to that observed for the Alg system and higher than the that observed for the Alg-DES hydrogel. These results are certainly due to the combined effect of both the drug incorporation, which increases the Young’s modulus, and the plasticizer effect of the DES, which strongly decreases this parameter, as observed in Figure 6a. In addition, for the compressive stress at 30%, the plasticizer effect of the DES is highlighted for both the Alg-DES and Alg-(DES + Ibu), which presented similar values to each other, but lower values than those of the other systems (Figure 6b). The Alg-(DES + Ibu) hydrogel presents more appealing mechanical properties for transdermal delivery purposes, since these mechanical properties translate into a loss of stiffness, allowing for a more pliable delivery system than Alg-Ibu to be obtained, enabling the hydrogel to better adapt to the wrinkles and deformations of the skin, and consequently allowing for better contact with the skin surface for drug permeation [52].

### 3.6. Dissolution and Permeation across Human Skin

The in vitro dissolution profile of ibuprofen was determined for the hydrogels loaded with ibuprofen solubilized in a slightly alkaline aqueous solution, or in the aqueous Arg:Gly solution. Before the dissolution tests, the incorporation efficiency of the drug in the hydrogels was calculated, and it was observed that both systems presented high incorporations of ibuprofen, viz. 82.5 ± 3.9% and 77.4 ± 2.3% for Alg-(DES + Ibu) and Alg-Ibu hydrogels, respectively. Since ibuprofen is commonly administered from 6 to 8 h [53], the dissolution assay was carried out in vitro for 8 h.

**Figure 7:**
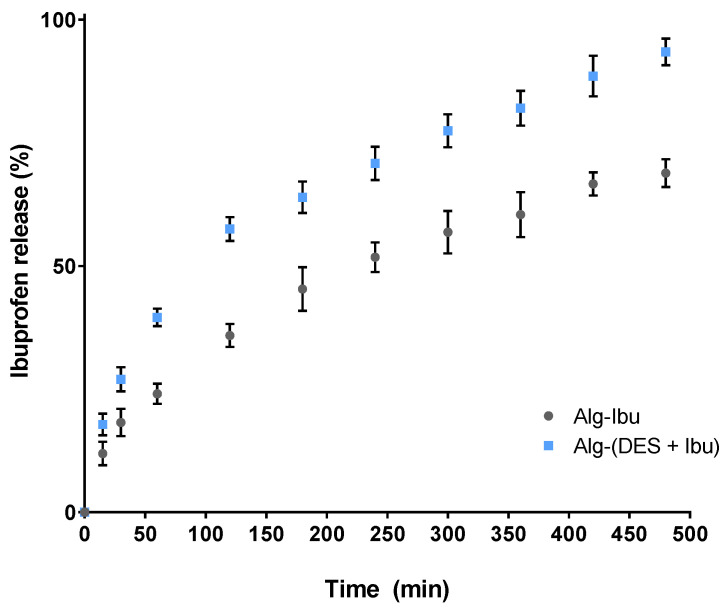
In vitro release profile of ibuprofen from alginate-based hydrogels in PBS solution. Profile data represented as mean ± SD of three independent experiments.

As depicted in Figure 7, ibuprofen shows a sustained dissolution profile for both systems; however, the dissolved amounts are higher for the hydrogels containing the DES solution with ibuprofen. After the first hour, the Alg-(DES + Ibu) hydrogel released 39.6 ± 1.5% of the total ibuprofen content, which represents a released amount that is 1.6-fold higher than that released by the Alg-Ibu system, with only 24.1 ± 1.7% released (Figure 7). After 8 h, the duration of the test, the Alg-(DES + Ibu) hydrogel released 93.5 ± 2.2% of the drug content, and the hydrogel Alg-Ibu only released 68.9 ± 2.5% (Figure 7). Indeed, for other, previously reported transdermal delivery systems [54,55] ibuprofen’s incomplete delivery represents an obstacle towards a more effective therapeutic outcome. For example, membranes of synthetic polymers, such as latex, prepared for the transdermal delivery of ibuprofen, released only 60% of the drug after 96 h [54]. These values can be enhanced when considering alternative formulations, such as nanoliposomes, which showed a drug release of 81–92% over a 24 h period [56]. However, the delivery period is still lengthy. The use of biopolymer-based membranes, such as bacterial cellulose, also enables a low release of ibuprofen to the media, achieving a plateau of 25–40% of total release within the first hour [44,55]. The development of more soluble forms of ibuprofen, such as ionic liquids, namely, cholinium ibuprofenate [44], or the drug conjugation with L-valine alkyl esters [55] can improve the drug release, especially when considering their incorporation into bacterial cellulose membranes. Cholinium ibuprofenate incorporation in bacterial cellulose allows for fast release of the drug, enabling 90% of release to be achieved within 10 min, which is beneficial for a fast therapeutic action [44]. The drug conjugation with L-valine alkyl esters also enables a high and faster delivery of the drug content, 87–90%, from 30 to 120 min, but does not necessarily translate into a higher permeation capacity and, therefore, into faster therapeutic action [55]. In this context, the Alg-(DES + Ibu) hydrogels developed in this work present a sustained release of the drug, with a high drug release, providing a fast and increasing drug dosage over the treatment period.

To infer if the improved dissolution profile of the hydrogels containing the DES solution translated into an effective drug permeation, the cumulative amount of ibuprofen that permeated from the two solid hydrogel systems on human epidermal skin was tested using a Franz diffusion cell. The cumulative mass of the drug, which permeated through the human skin samples after 6 h at 32 °C, is presented in Table 1. In terms of absolute quantities, after 6 h, ibuprofen permeated to a higher extent from the Alg-(DES + Ibu) hydrogel than from the Alg-Ibu (382.4 ± 16.6 µg∙cm^−2^ vs. 44.8 ± 15.7 µg∙cm^−2^). The presence of the DES aqueous solution comprising ibuprofen in the alginate hydrogel allowed for an 8.5-fold increase in the amount of ibuprofen that permeated through human epidermis. These results are in total agreement with the previously described dissolution profiles.

The amount of ibuprofen that permeated from the Alg-Ibu hydrogel is in accordance with the values obtained for commercial ibuprofen gels (≈40 µg∙cm^−2^) for 6 h of permeation [57]. The incorporation of this model drug in biopolymer-based systems, such as bacterial cellulose membranes, has been proven to enhance the transdermal delivery of the drug when compared to commercial gel formulations [58]. The amount permeated achieved using the DES solution comprising ibuprofen in alginate-based hydrogels is not only considerably higher (8.5-fold) than the amount obtained for commercial formulations (≈40 µg∙cm^−2^) and for other biopolymer-based systems, but also comparable to the values that were observed for formulations of ibuprofen conjugated with L-valine alkyl esters, which also display high drug solubility (382.35 ± 1.05 µg∙cm^−2^) [59]. The Alg- (DES + Ibu) hydrogels reported herein enable higher permeation enhancements than those previously reported when using DES formulations that comprised the drug and a permeation enhancer, such as menthol (ibuprofen:menthol) [60]. However, the previously mentioned values are commonly evaluated for a 24-h assay period. The Alg-(DES + Ibu) hydrogels presented herein provided higher permeated amounts after only 6 h, allowing for a faster therapeutic onset to be obtained than that of typical delivery systems.

## 4. Conclusions

DES aqueous solutions comprising ibuprofen were studied in this work, aiming to improve the drugs’ characteristics and their transdermal delivery when using an alginate hydrogel. Herein, we demonstrate that Arg:Gly aqueous solutions are able to improve the solubility of ibuprofen up to 7917-fold (solution with 60% *w*/*w* of the DES) at room temperature, providing competitive pharmaceutical co-solvents to those commonly applied in pharmaceutical formulations. Furthermore, this DES aqueous solution is able to preserve the ibuprofen’s stability even when the drug is submitted to higher room temperatures (up to 37 °C over 1 month), allowing for their storage in non-refrigerated conditions. Moreover, these formulations showed non-toxicity towards macrophages, and slightly increased the therapeutic action of the drug (namely, its anti-inflammatory activity).

The combination of these DES aqueous solutions and an alginate-based hydrogel resulted in efficient drug-delivery systems for the transdermal administration of ibuprofen. Based on the results, the plasticizer effect of the Arg:Gly DES can be highlighted, which allowed for hydrogel systems with a more homogenous and defined structure to be obtained, which are more flexible when handled. Moreover, the dissolution of the drug from the biopolymer-based system with the Arg:Gly solution shown a sustained release of the drug, achieving 93.5 ± 2.2% of drug release after 8 h. Finally, the permeation of the drug through the human skin was improved by up to 8.5-fold when the Ag:Gly aqueous solutions comprising ibuprofen was used, highlighting the successful partnership between DES and alginate hydrogels for the transdermal delivery of this drug. Moreover, this study forms a basis for future optimizations of the developed systems and additional studies using different drugs, DES and polymeric systems, with the aim of improving the transdermal administration of distinct drugs. In advanced stages of this research, in vivo studies will also be considered, aiming to guarantee a safe and efficient administration of these systems and confirm their potential as a strategy for use in different clinical scenarios.

## Figures and Tables

**Table 1:** The average cumulative mass of ibuprofen after 6 h permeation from the hydrogel with ibuprofen and the hydrogel with ibuprofen solubilized in the DES aqueous solution test across human epidermis.

Sample	Cumulative Mass (µg Ibu∙cm^−2^)
Alg-Ibu	44.81 ± 15.71
Alg-(DES + Ibu)	382.43 ± 16.62

## Data Availability

Not applicable.

## Supplementary Materials

The following supporting information can be downloaded at: https://www.mdpi.com/article/10.3390/pharmaceutics14040827/s1, Figure S1: (**a**) ^1^H NMR and (**b**) ^13^C NMR spectra of arginine:glycerol DES in D_2_O; Figure S2: FTIR-ATR spectra of glycerol, arginine and the DES arginine:glycerol. Table S1: Ibuprofen solubility in aqueous solutions of arginine:glycerol (1:4) at 25 and 37 ºC, expressed in mg∙mL^−1^ and mol∙dm^−3^ (M). Results expressed as mean ± SD of independent measurements.
