# Supplementary material for: Deep Eutectic Solvent Formulations and Alginate-Based Hydrogels as a New Partnership for the Transdermal Administration of Anti-Inflammatory Drugs
Source: Pharmaceutics. 2022 Apr 10;14(4):827. doi: 10.3390/pharmaceutics14040827 (PMC9031671; doi:10.3390/pharmaceutics14040827)
Supplement: Supplementary file 1 [file pharmaceutics-14-00827-s001.zip › pharmaceutics-1646792-supplementary.pdf]

pharmaceutics

Supplementary Information

Article

Deep eutectic solvent formulations and alginate-based hydrogels as a new partnership for the transdermal administration of anti-inflammatory drugs

Sónia N. Pedro [1], Maria S. M. Mendes [1], Bruno M. Neves [2], Isabel Filipa Almeida [3,4], Paulo Costa [3,4], Inês Correia-Sá [5], Carla Vilela [1], Mara G. Freire [1,*], Armando J. D. Silvestre [1] and Carmen S. R. Freire [1,*]

[1] Department of Chemistry, CICECO-Aveiro Institute of Materials, University of Aveiro, 3810-193 Aveiro, Portugal; soniapedro@ua.pt (S.N.P.); msilvinamm@ua.pt (M.S.M.M.); cvilela@ua.pt (C.V.); armsil@ua.pt (A.J.D.S)
[2] Department of Medical Sciences and Institute of Biomedicine – iBiMED, University of Aveiro, 3810-193 Aveiro, Portugal; bruno.neves@ua.pt (B.M.N.)
[3] Associate Laboratory i4HB - Institute for Health and Bioeconomy, Faculty of Pharmacy, University of Porto, 4050-313 Porto, Portugal; ifalmeida@ff.up.pt (I.F.A.); pccosta@ff.up.pt (P. C.)
[4] UCIBIO/REQUIMTE, MedTech, Laboratory of Pharmaceutical Technology, Department of Drug Sciences, Faculty of Pharmacy, University of Porto, 4050-313 Porto, Portugal;
[5] Department of Plastic, Aesthetic, Reconstructive and Aesthetic Surgery, Centro Hospitalar de S. João, 4200-319 Porto, Portugal

* Correspondence: maragfreire@ua.pt (M.G.F.); cfreire@ua.pt (C.S.R.F.)

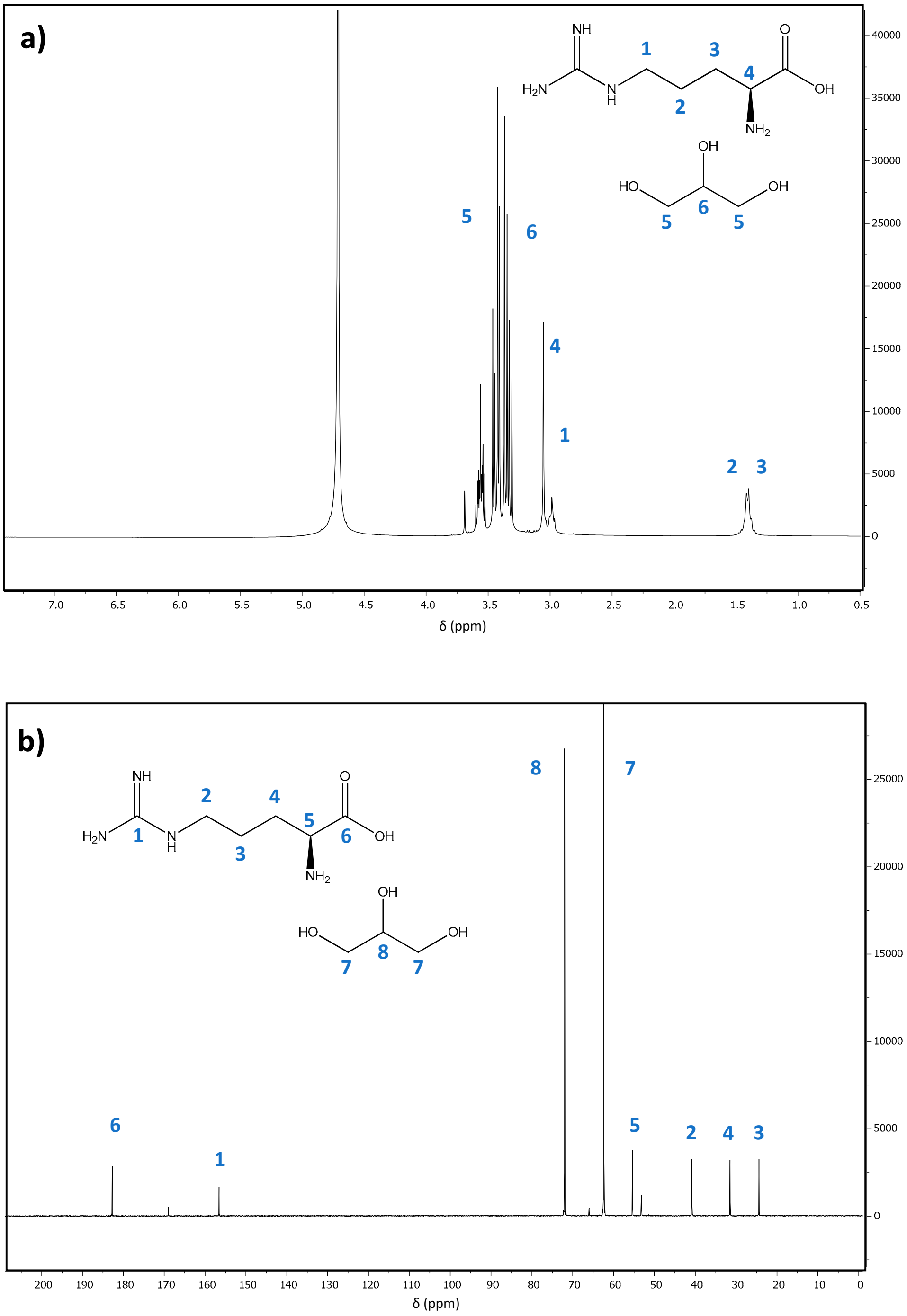

Figure S1. (a) ^{1}H NMR and **(b)** ^{13}C NMR spectra of arginine:glycerol DES in D_2O.

Arginine:glycerol: ^{1}H NMR, δ/ppm (300 MHz, D_2O): 1.41 [4 H, m, CH_2(2,3)]; 2.98 [2 H, m, CH_2(1)]; 3.05 [1 H, m, CH_2(4)]; 3.39 [1 H, m, CH_2(6)]; 3.55 [4 H, m, CH_2(5)]. ^{13}C NMR, δ/ppm (75.47 MHz, D_2O): 24.40 [CH_2(3)]; 31.50 [CH_2(4)]; 40.86 [CH_2(2)]; 53.21 [CH(5)]; 62.15 [CH_2(7)]; 71.97 [CH(8)]; 156.57 [C=NH(1)]; 182.54 [C=O(6)].

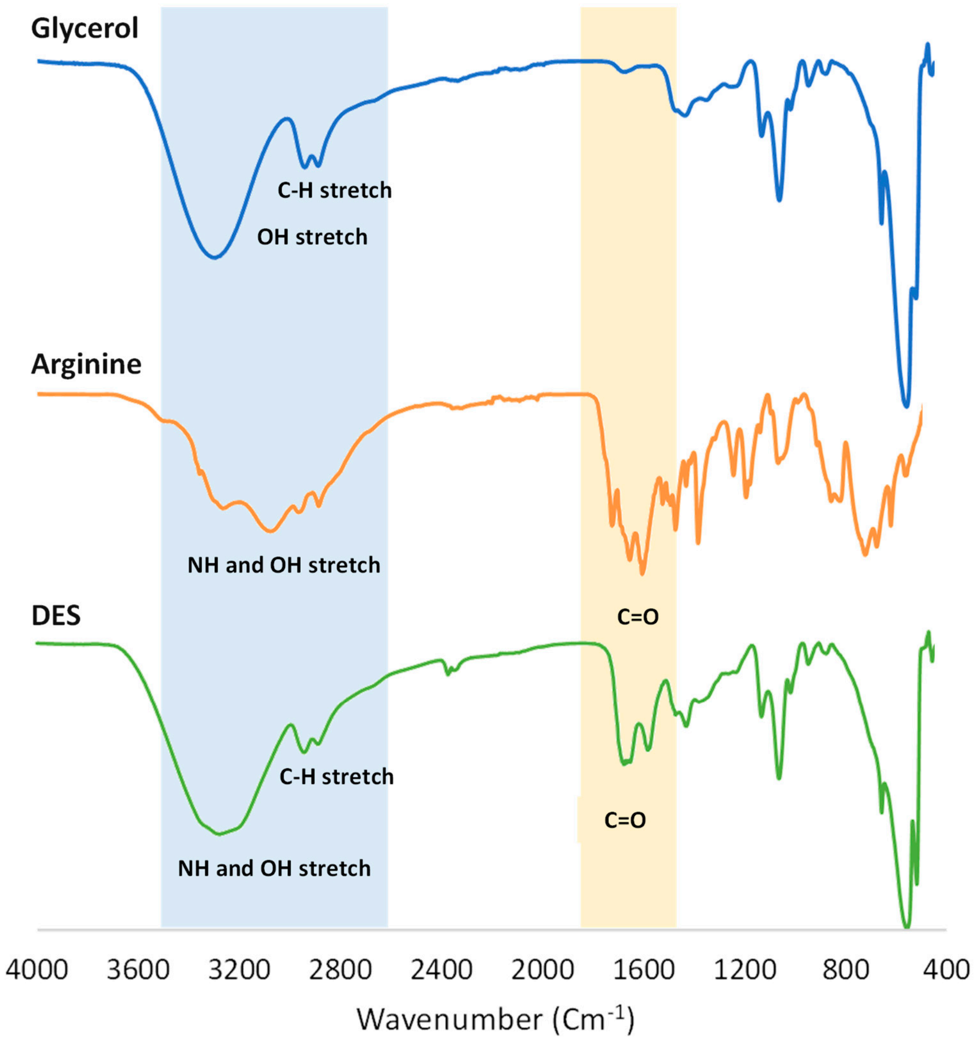

Figure S2. FTIR-ATR spectra of glycerol, arginine and the DES arginine:glycerol.

Table S1. Ibuprofen solubility in aqueous solutions of arginine:glycerol (1:4) at 25 and 37 ºC, expressed in $mg \cdot mL^{-1}$ and $mol \cdot dm^{-3}$ (M). Results expressed as mean ± SD of independent measurements.

	25 ºC		37 ºC	
**% of DES in water (w/w)**	$mg \cdot mL^{-1}$	M	$mg \cdot mL^{-1}$	M
0	$(5.926 \pm 0.433) \times 10^{-3}$	$(2.163 \pm 0.158) \times 10^{-5}$	$(8.731 \pm 0.484) \times 10^{-3}$	$(3.186 \pm 0.177) \times 10^{-5}$
10	$(7.387 \pm 0.346) \times 10^{0}$	$(2.696 \pm 0.126) \times 10^{-2}$	$(8.144 \pm 0.131) \times 10^{0}$	$(2.972 \pm 0.048) \times 10^{-2}$
20	$(1.558 \pm 0.012) \times 10^{1}$	$(5.688 \pm 0.042) \times 10^{-2}$	$(1.597 \pm 0.017) \times 10^{1}$	$(5.830 \pm 0.060) \times 10^{-2}$
30	$(1.725 \pm 0.034) \times 10^{1}$	$(6.296 \pm 0.124) \times 10^{-2}$	$(2.433 \pm 0.041) \times 10^{1}$	$(8.881 \pm 0.149) \times 10^{-2}$
40	$(2.367 \pm 0.083) \times 10^{1}$	$(8.638 \pm 0.302) \times 10^{-2}$	$(2.975 \pm 0.083) \times 10^{1}$	$(1.086 \pm 0.030) \times 10^{-1}$
50	$(2.459 \pm 0.048) \times 10^{1}$	$(8.973 \pm 0.177) \times 10^{-2}$	$(3.604 \pm 0.070) \times 10^{1}$	$(1.315 \pm 0.026) \times 10^{-1}$
60	$(4.692 \pm 0.046) \times 10^{1}$	$(1.712 \pm 0.017) \times 10^{-1}$	$(4.977 \pm 0.084) \times 10^{1}$	$(1.816 \pm 0.031) \times 10^{-1}$
